# Uncommon abdominal “egg-shelled” lesions mimic hepatic echinococcosis: Two cases report

**DOI:** 10.3389/fsurg.2022.944980

**Published:** 2022-09-09

**Authors:** Yubo Liao, Guo Zhou, Chong Yang, Yu Zhang

**Affiliations:** ^1^ School of Medicine, Chengdu Medical College, Chengdu, China; ^2^ Ultrasonography Department, Sichuan Provincial People’s Hospital, University of Electronic Science and Technology of China, Chengdu, China; ^3^ Clinical Immunology Translational Medicine Key Laboratory of Sichuan Province / Organ Transplantation Center, Sichuan Provincial People’s Hospital, University of Electronic Science and Technology of China, Chengdu, China; ^4^ Department of Hepatobiliary and Pancreas Surgery, Sichuan Provincial People’s Hospital, University of Electronic Science and Technology of China, Chengdu, China

**Keywords:** “egg-shelled” lesions, hepatic echinococcosis, diagnosis, treatment, surgery

## Abstract

**Background:**

Primary abdominal “egg-shelled” lesions with positive anti-echinococcus IgG antibody were misdiagnosed as echinococcosis.

**Case presentation:**

Herein we report two cases with primary abdominal “egg-shelled” lesions were misdiagnosed as echinococcosis. **Case 1:** A 44-year-old woman presented to our department with a history of slight abdominal pain for 4 months. After admission, the laboratory tests indicated a positive anti-echinococcus IgG antibody status. The contrast-enhanced CT scan showed a 12 × 12 cm “me contrast-mass located in the hepatorenal area. The patient had the entire mass and the right adrenal gland resected. This patient recovered smoothly and was discharged uneventfully 20 days after the operation. The pathologic diagnoses was adrenal lymphangioma. **Case 2:** A 55-year-old woman was admitted with a history of an abdominal mass for over 10 years. After admission, her anti-echinococcus IgG antibody was positive. The contrast-enhanced CT scan revealed a heterogeneous, solid mass measuring 10 × 9 × 8 cm in the right hepatic lobe. A laparoscopic exploration was performed, and the surgery revealed that the mass arose from the retroperitoneal tissue rather than the liver. Finally, the pathologic diagnoses were paraganglioma with necrosis and cystic changes.

**Conclusion:**

Enhanced CT scan and contrast-enhanced ultrasound scans are important for distinguishing echinococcosis disease from the other “egg-shelled” lesions. Surgical resection is the main treatment method for this disease. Minimally invasive surgery is recommended but should be performed by experienced surgical teams. Immunohistochemical examination is important for the pathologic diagnosis.

## Background

The “egg-shelled” lesion is a mass with an eggshell-like calcified wall, characterized by homogeneous intracapsular necrotic material, that will not or will only be slightly enhanced on contrast-enhanced computed tomography (CT) scans. Many primary lesions could present as egg-shelled lesions with calcification including retroperitoneal pseudocyst, adrenal tuberculosis, pelvic schwannoma, and acute calcific retropharyngeal tendinitis (1–5). As a heterogeneous primary disease, an abdominal “egg-shelled” mass is easily misdiagnosed as echinococcosis based on the similar imaging findings (6, 7).

Echinococcosis is an aggressive and potentially lethal zoonotic infection that often occurs in the hepatic lobe(s) (8). Based on the different diseases associated with Echinococcus, human echinococcosis can be divided into two types: cystic echinococcosis (CE, caused by *Echinococcus granulosus*) and alveolar echinococcosis (AE, caused by *Echinococcus multilocularis*) (9). However, egg-shelled calcifications may be present in the imaging of CE or AE cases and many other diseases. The geographical history and positive anti-echinococcus IgG antibody status also can mislead the diagnosis (10).

Herein, we present two cases that had “egg-shelled” lesions that were misdiagnosed as hepatic echinococcosis. As abdominal “egg-shelled” masses are rare and can be present in different diseases, including echinococcosis, the purpose of this paper is to present our experience with treatment and to reduce the misdiagnosis rate. This work was supported by the Ethics Committee of Sichuan Provincial People's Hospital.

## Case presentation

### Case 1

A 44-year-old woman presented to our department with a history of slight abdominal pain for 4 months. She had a long history of living in an area with echinococcosis, and there was no significant familial medical history for this patient. The medical examination found a mass located in the middle right abdominal region with tenderness and unclear boundaries.

**Figure 1:**
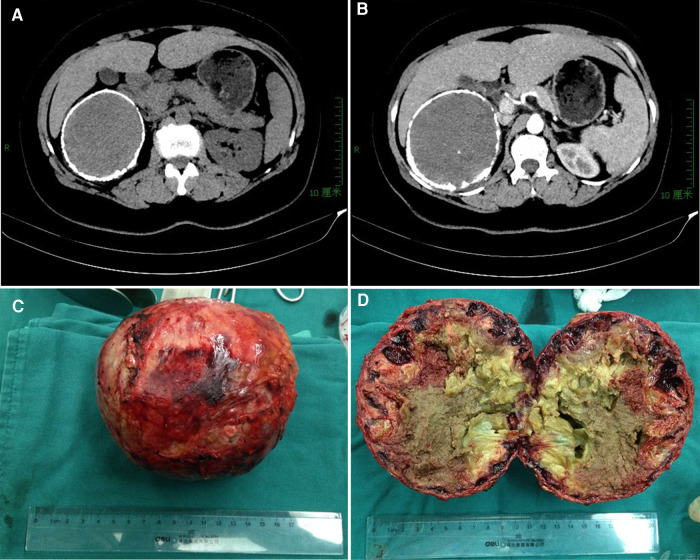
Ct imaging and specimen for case 1. (**A**) an “egg-shell” mass locating in the right subhepatic area; (**B**) mild enhanced on contrast-enhanced CT scans for the mass in the artery phase; (**C**) the resected specimen with a calcific shell; (**D**) homogeneous necrotic content with point calcifications.

After admission, the laboratory tests indicated normal white blood cell (WBC) [4.74 × 10^9^/L, (references 3.50–9.5 × 10^9^/L)] and absolute eosinophil counts [0.45 × 10^9^/L, (references 0.02–0.52 × 10^9^/L)], the serum tumor markers were normal and a positive anti-echinococcus IgG antibody status. The contrast-enhanced CT scan showed a 12 × 12 cm “egg-shelled” mass located in the hepatorenal area (Figure 1A). This mass had a well-distributed calcified wall wrapping around the homogeneous content with point calcifications. The content showed mild enhancements during arterial phases (Figure 1B). The primary diagnosis was abdominal AE disease. This patient underwent laparotomy surgery for a mass resection. During surgery, we found a large calcified mass located near the upper right kidney arising from the right adrenal gland adjacent to the lower right hepatic lobes. The patient had the entire mass and the right adrenal gland resected. This patient recovered smoothly and was discharged uneventfully 20 days after the operation.

The resected specimen had a calculated shell (Figure 1C), which wrapped around the homogeneous necrotic content with point calcifications (Figure 1D). The pathological examination revealed calcification of the surface, necrosis and sclerotization of the contents. The immunohistochemical examination revealed positive expressions of CD-34, CD-31 and D2–40 but no expression of CK. These findings supported a pathologic diagnosis of adrenal lymphangioma with hemorrhage and hematoma formation. There were no significant findings in the follow-up 6 years after surgery for this patient.

### Case 2

**Figure 2:**
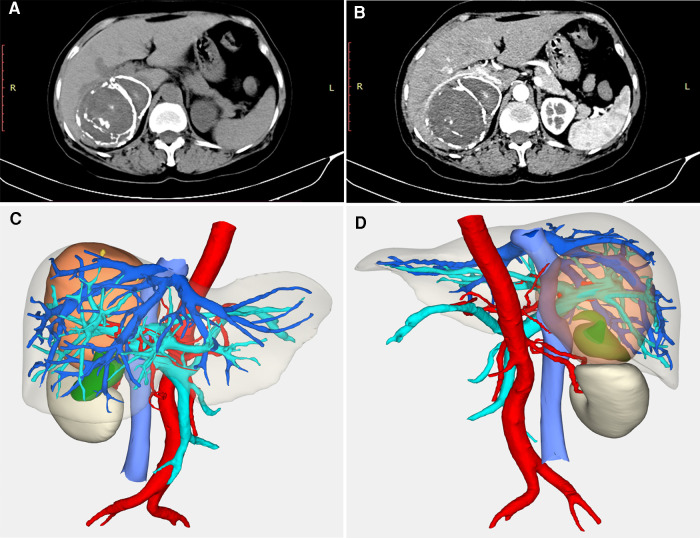
Imaging examination for case 2. (**A**) an “egg-shell” mass locating in the right subhepatic area; (**B**) the mass had a calcified edge wrapping around the homogeneous content with multipoint calcifications and calcified separations in the artery phase; (**C**) three-dimensional CT reconstructed imaging revealed that the right hepatic vein and hepatic pedicle were compressed from the dorsal to the ventral part (anterior); (**D**) three-dimensional CT reconstructed imaging (posterior).

A 55-year-old woman was admitted with a history of an abdominal mass for over 10 years. The patient had unremarkable medical and family histories, and her physical examination was normal. After admission, the laboratory evaluations indicated normal WBC (7.25 × 10^9^/L) and absolute eosinophil counts (0.32 × 10^9^/L), and the other significant index were negative, except her serum anti-echinococcus IgG antibody was positive. The contrast-enhanced CT scan revealed a heterogeneous, solid mass measuring 10 × 9 × 8 cm in the right hepatic lobe (Figure 2A). The mass had a clearly calcified edge wrapping around the homogeneous content with multipoint calcifications and calcified separations (Figure 2B). Furthermore, three-dimensional CT reconstructed imaging revealed that the right hepatic vein and hepatic pedicle were involved from the dorsal to the ventral part but were not invaded into or encapsulated (Figures 2C,D). The primary diagnosis was hepatic CE (CE-5 type).

**Figure 3:**
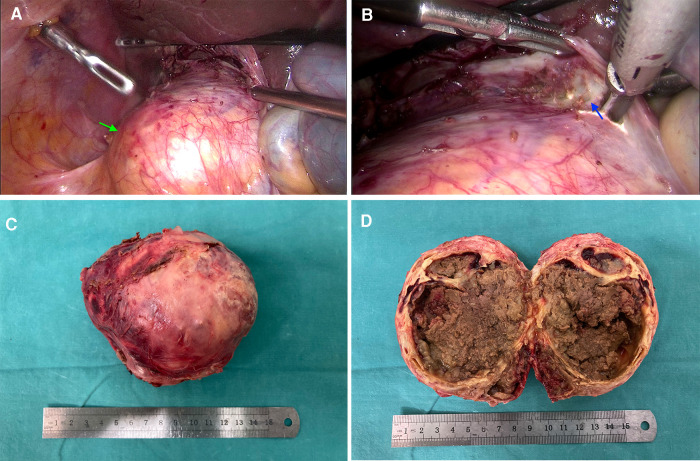
Laparoscopic exploration and specimen for case 2. (**A**) an “egg-shell” mass (green arrow) arose from the retroperitoneal tissue; (**B**) there is a clear edge between the mass and the hepatic lobes (blue arrow); (**C**) the resected specimen with a calcific shell; (**D**) homogeneous necrotic content with point calcifications and calcified separations.

After an appropriate preoperative preparation, a laparoscopic exploration was performed, and the surgery revealed that the mass arose from the retroperitoneal tissue rather than the liver. There was an obvious calcification of this mass which was pressing against the right kidney and lower right hepatic lobes (Figure 3A), the edge of which was clear (Figure 3B). A laparoscopic retroperitoneal mass resection was successful, and the patient recovered smoothly and was discharged uneventfully 7 days after the operation.

It was found that the resected mass had a calcified shell (Figure 3C), which wrapped around the bleeding necrotic content with point calcifications and calcified separations (Figure 3D). During a microscopic exam, we found fibroplasia and residual cell components that expressed neuroendocrine markers. The immunohistochemical examination revealed positive expressions of syn, HMB45, CD56, CD68 (weak) and CK (weak) but no expression of SALL4. The percent for Ki-67 was 2%. These findings supported a pathologic diagnosis of paraganglioma with necrosis and cystic changes.

## Discussion and conclusions

**Figure 4:**
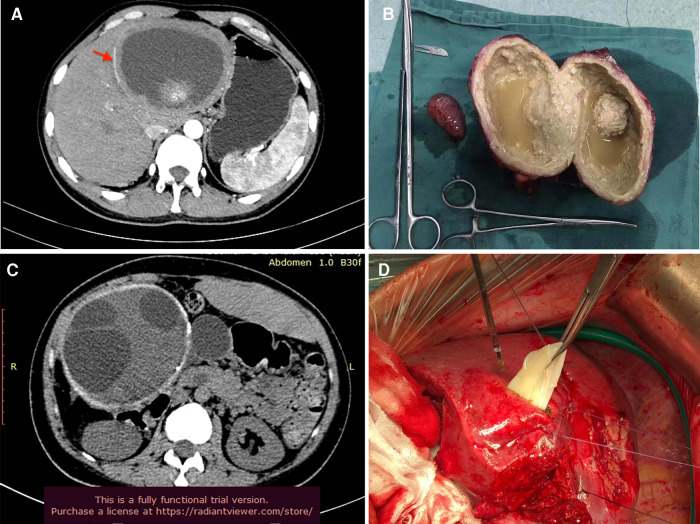
Typical AE and CE with eggshell-like calcification. (**A**) AE lesion with “egg-shelled” calcification (red arrow); (**B**) the resected AE lesion; (**C**) CE lesion with “egg-shelled” calcification and multi-cysts; (**D**) the cysts of CE during surgery.

Diseases with “egg-shelled” calcified lesions on imaging are heterogeneously. To date, some primary occurred lesions with typically “eggshell-like” calcification had been reported (1–5, 11). Some infectious disease, including AE and CE disease, also presented “egg-shelled” calcification for some special cases. Based on our experience, hepatic AE disease may present with large “egg-shelled” masses because of the homogeneous intracapsular liquefied necrotic material based on some published research (12) and even our experience (Figures 4A,B). At the same time, it's well known that calcified CE lesions (type-5) present as an “eggshell-like” mass (13). Also, some CE cases with “egg-shelled” wall were found during our surgical exploration (Figures 4C,D). if the patient has been living for a significant time in an area with echinococcosis and/or is positive for anti-echinococcus IgG antibodies, there may be confusion regarding the diagnosis (10).

There are some common characteristics of the “eggshell like” mass. First, most of the “egg-shelled” lesions are benign rather than malignant. For instance, *Imisairi* et al reported an adrenal tuberculosis case with eggshell-like calcifications (4). Hepatic CE and AE diseases are conventionally deemed benign despite whose infiltrative growth features. Second, most cases present as asymptomatic or have vague blunt abdominal pain such as these 2 cases. It is assumed that the time for the formation of the calcified shell is very long, and most of the egg-shelled lesion cases conventionally have a long-term history. Third, the relationship between most “egg-shelled” lesions and the adjacent organs is with compressing or by adhering rather than by infiltrating. Therefore, a radical resection is possible for most cases without the need to open the calcified cyst.

Accurate preoperative diagnoses in “egg-shelled” lesions are difficult. This challenge is caused by the similar imaging characteristics, including the presence of a calcified shell and a low-density inner cyst necrotic material. The main reason for the misdiagnoses of the 2 cases presented here is the similar CT findings as in hepatic echinococcosis. Additionally, the positive anti-echinococcus IgG antibody statuses and the geographical histories of the patients misdirected the diagnoses. The positive anti-echinococcus IgG antibody statuses are caused by the antigenic stimulus by the echinococcus when the patients lived in the disease area, rather than by actual hepatic echinococcosis disease (10).

It is important to distinguish echinococcosis lesions from other “egg-shelled” lesions because the overflow of the inner cyst liquid may cause an extensive abdominal hydatid metastasis in CE. Additionally, the overflow of necrotic material in AE lesions could induce an extended abdominal infection. Enhanced CT scans are the most important imaging examinations for distinguishing these tumors. For CE with a calcified shell, many subcysts are often present on imaging. However, the subcysts should not be assumed to be multi-internal septations, such as cystadenomas. On the other hand, AE lesions often have a thick calcified shell, unsmooth inner walls and a homogeneous cyst with necrotic inner material. In addition to CT scans, contrast-enhanced ultrasound scans (CEUS) are also important for distinguishing echinococcosis disease from others. In advanced AE, the cystic components are comprised of metacestodal vesicles and have liquefactive necrosis; the solid components have coagulation necrosis and calcifications with no or little microcirculation. After the injection of a contrast agent, the CEUS shows a nonenhancement, similar to the black hole effect (14). At the same time, AE or CE lesions often occur in the liver rather than in other organs or tissues.

Radical surgical resection is needed for both echinococcosis and other “egg-shelled” lesions. Laparoscopic surgery is a safe technique for radical resection and has been utilized for years. However, if the adjacent tissue (such as the right adrenal gland for the case 1) is easily to be touched during laparoscopic surgery causing serious consequences, laparoscopic mass resection should be recommended. At the same time, the opening of “egg-shelled” cysts for pressure reduction is not recommended by previously reported cases. For some CE cases presented as Figure 4C,D, some activated hydatid eggs may cause abdominal metastasis. For case 2, we successfully performed a laparoscopic surgical resection without opening the cyst. This minimally invasive surgery depends on the experience of the surgical team for a positive outcome in cases suspected of echinococcosis to avoid interoperative hydatid metastasis or abdominal infections. The pathologic diagnosis depends upon the immunohistochemical examination, which determines the future treatment.

In conclusion, “egg-shelled” lesions are rare diseases, and most of them are benign diseases mimicking hepatic echinococcosis. Accurately differentiating nonparasitic lesions from echinococcosis disease is important for surgical planning. Surgical resection is the main method for the treatment of “egg-shelled” lesions. Minimally invasive surgery is recommended and should be performed by experienced surgical teams. Immunohistochemical examination is important for the pathologic diagnosis.

## Data Availability

The original contributions presented in the study are included in the article/Supplementary files, further inquiries can be directed to the corresponding author/s.
